# A new genus and two new species of Southeast Asian Bidessini as well as new synonyms for Oceanian species (Coleoptera, Dytiscidae)

**DOI:** 10.3897/zookeys.647.11231

**Published:** 2017-01-30

**Authors:** Michael Balke, Johannes Bergsten, Liang-Jong Wang, Lars Hendrich

**Affiliations:** 1 SNSB-Zoologische Staatssammlung München, Münchhausenstrasse 21, München, Germany; 2 Department of Zoology, Swedish Museum of Natural History, Box 50007, SE-10405 Stockholm, Sweden; 3 Division of Forest Protection, Taiwan Forestry Research Institute, Taipei, Taiwan

**Keywords:** Bidessini, Dytiscidae, new combination, new genus, new species, new synonymy, Oceania, Southeast Asia

## Abstract

*Rompindessus jenisi* Balke, Bergsten & Hendrich, **gen. n.** et **sp. n.** is described from near Rompin village in West Malaysia. The new genus is characterized by the presence of an occipital line and basal pronotal striae, the presence of a thick anterior bead on clypeus and two-segmented parameres as well as by the absence of basal elytral striae, the absence of sutural line on elytron, the absence of basal epipleural transverse carina, and the absence of longitudinal elytral carina. Moreover, male pro- and mesotarsus appear stout, and distinctly dilated laterally; the pronotum is comparably long and parallel-sided and the colour of beetle conspicuous dark orange. *Leiodytes kualalipis* Balke, Wang, Bergsten & Hendrich, **sp. n.** is described from West Malaysia (Pahang) and South Vietnam (Cat Tien). It is well characterized by its large size, elongate body and the form of the median lobe. *Limbodessus fijiensis* (J. Balfour-Browne, 1944), **comb. n.** described from Fiji is a new synonym of *Limbodessus curviplicatus* (Zimmermann, 1927) described from Samoa.

## Introduction

There are currently 48 genera in the Bidessini (Nilsson 2016; Miller 2016; Miller and Bergsten 2016). With a body length of typically around 1–3 mm, they constitute most of the smaller species of Dytiscidae. Bidessini genera have to date been justified mainly on a diagnostic combination of structural features (Biström 1988; Balke et al. 2002; Miller and Short 2015; Miller and Bergsten 2016) rather than apomorphies and this had to lead to recognition of genera that render other paraphyletic (Balke and Ribera 2004). Some of these features such as presence / absence of elytral striae (plicae) or occipital line have been shown to vary within clades of closely related species (Balke et al. 2015) or even within one species (Balke unpublished). In this context, the use of phylogenetic reconstructions based on DNA sequence data offers a source of information that helps to delineate monophyletic entities (Hendrich and Balke 2009; Balke et al. 2013). Here, we report the discovery of two new species of Bidessini from Peninsula Malaysia. Both species are known from older collections only and therefore we use morphological characters and a pragmatic approach to make tentative generic assignments that lead us to suggest one new genus here.

## Material and methods

Drawings of the male genitalia were made based on digital images. The beetles were studied with a Leica MZ 12.5 stereomicroscope at 10–100x and a Scanning Electron Microscope (JSM-5600. JOEL, Tokyo, Japan) at 90–1000X. The terminology to denote the orientation of the genitalia follows Miller and Nilsson (2003). Label data of the type material are cited in quotation marks. The following abbreviations were used in the text: TL (total length), TL-H (total length without head), and MW (maximum width). Google Earth (http://earth.google.com) was used to locate localities and the coordinates are given in decimal degree format. Specimens mentioned in this work are deposited in several collections, which are abbreviated in the text as follows:

BMNH
Natural History Museum [former British Museum (Natural History)], London, England 


CSR
 Collection Saverio Rocchi, Firenze, Italy 


CWT
 Collection L. J. Wang, Taipei, Taiwan 


MNHN
Muséum National d’Histoire Naturelle, Paris, France 


NHMW
 Naturhistorisches Museum Wien, Austria 


TFRI
Taiwan Forestry Research Institute, Taipei, Taiwan 


ZSM
Zoologische Staatssammlung München, Germany 


## Taxonomy

### Rompindessus

Taxon classificationAnimaliaColeopteraDytiscidae

Balke, Bergsten & Hendrich gen. n.

http://zoobank.org/2562EA40-B2CA-4909-89B4-8332C6367AE0

#### Type species.

*Rompindessus jenisi* sp. n. by present designation.

#### Diagnosis.

Of the set of structural features generally used to classify Bidessini, the following combination is present in this taxon: 1) occipital line present; 2) basal pronotal striae present; 3) basal elytral striae absent; 4) sutural line on elytron absent; 5) basal epipleural transverse carina absent; 6) clypeus with thick anterior bead (or margin); 7) longitudinal elytral carina on disc absent; and 8) parameres two-segmented. Moreover, the male pro- and mesotarsus appear stout, and distinctly dilated laterally; the pronotum is comparably long and parallel-sided and the colour of beetle is conspicuously dark orange. This differentiates *Rompindessus* gen. n. from all other Bidessini. In the key to Bidessini genera by Miller and Bergsten (2016) *Rompindessus* keys out to *Platydytes* Biström, 1988, which only occurs in sub-Saharan Africa. Apart from geography, *Rompindessus* can be distinguished from *Platydytes* by the thickly bordered clypeal margin (finely bordered to indistinct in *Platydytes*), the enlarged, laterally expanded male pro- and mesotarsomeres I-III, the dark orange colouration with dark speckles on elytra, and the more discontinuous body outline between pronotum and elytra.

#### Etymology.

Named after the collecting locality, Rompin village, and the ending – dessus as used for many genera in Bidessini.

### Rompindessus jenisi

Taxon classificationAnimaliaColeopteraDytiscidae

Balke, Bergsten & Hendrich sp. n.

http://zoobank.org/73B2A0D7-62AC-4891-9510-3504A50D96EC

#### Holotype.

Male (NHMW). “Malaysia, Pahang, 40km W Rompin, Selendang, 29.4.–6.6., leg. I. Jenis, 1993”.

#### Type locality.

Situated around Malaysia, Pahang, Selendang, between these points 2.622516°N 103.334934°E / 2.609909°N 103.382443°E / 2.643574°N 103.409337°E, it is not known where exactly this particular beetle was discovered. The altitude is around 50 to 250m (Jeniš pers. comm.). According to satellite images in Google Earth, the lower elevations are now heavily transformed into oil palm plantations.

#### Description.

Habitus elongate oval. Measurements: TL = 2.45 mm, TL-H = 2.15 mm; MW = 1.1 mm; elytra 3.5× longer than pronotum.

**Figure 1.**
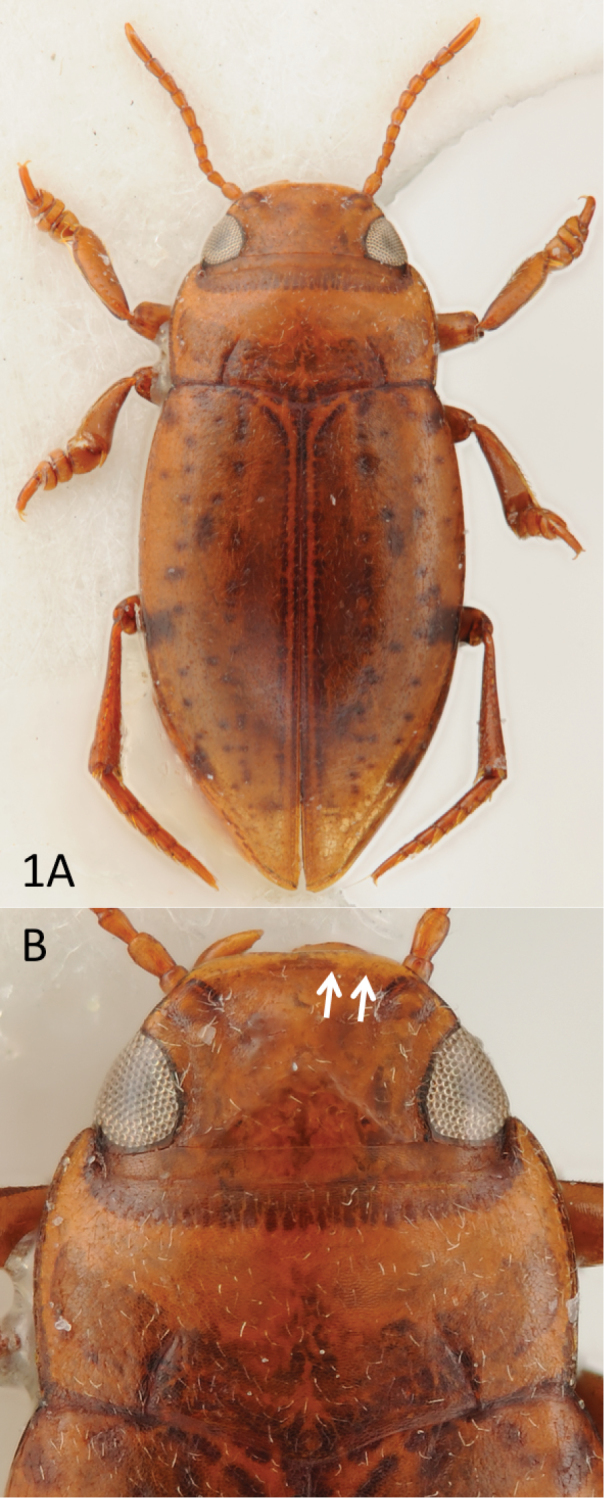
*Rompindessus jenisi* gen. et sp. n., holotype, habitus (**A**), head, pronotum and base of elytra (**B**). White arrows point to the thick anterior bead of clypeus.

**Colouration.** Head, pronotum and elytron dark orange, the latter with few darker speckles (Fig. 1). Ventral side and appendages testaceous.

**Surface sculpture.** Head with distinct microreticulation posterior of occipital line; finer microreticulation along eyes and clypeus, frons without microreticulation and thus shiny / polished but with few setiferous punctures. Pronotum and elytron with distinct microreticulation and sparse setiferous punctation. Ventral surfaces polished, with faint and sparse setiferous punctation.

**Structures.** Head with distinct occipital line and broadly beaded clypeus (Fig. 1A). Pronotum with faint lateral bead and distinct basal striae, the latter are curved inwards (Fig. 1). Elytron without basal striae and without sutural line. Basal epipleural transverse carina absent. Metathoracic wings apparently fully developed (not dissected but seen from opening caused by previous removal of the last three ventrites). Pro- and mesotarsus appearing stout as they are distinctly dilated laterally.

**Figure 2.**
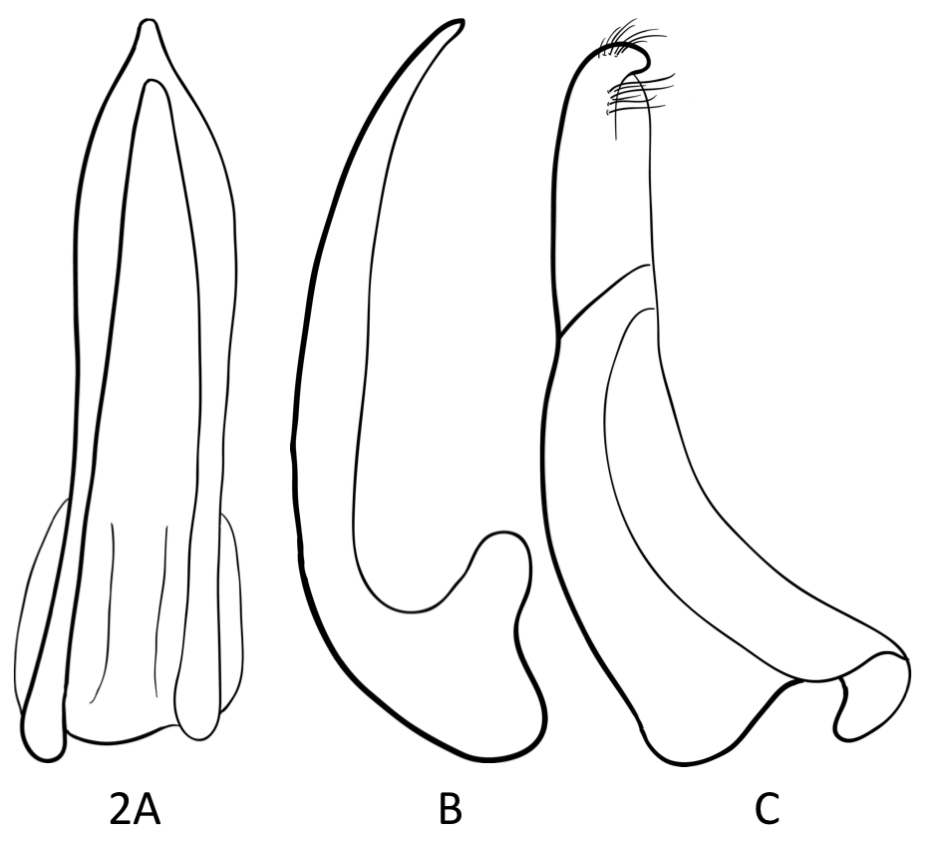
*Rompindessus jenisi* gen. et sp. n., holotype, median lobe of aedeagus, ventral view (**A**), lateral view (**B**), paramere, lateral view (**C**).

**Male.** Median lobe of aedeagus simply curved (Fig. 2A, B), lateral lobes (parameres) of two parts and of general Bidessini type (e.g. as figured for *Uvarus lacustris* (Say, 1823), *Leiodytes evanescens* (Boheman, 1848), *Platydytes incognitus* Biström, 1988 and *Bidessus unistriatus* (Goeze, 1777) in Biström (1988)) (Fig. 2C).

**Female.** Unknown.

#### Etymology.

Named after Ivo Jeniš, discoverer of this species. The specific epithet is a substantive in the genitive case.

#### Distribution.

Only known from the type locality.

#### Habitat.

Unknown.

### Leiodytes

Taxon classificationAnimaliaColeopteraDytiscidae

Guignot, 1936

#### Type species.

*Hydroporus evanescens* Boheman, 1848

#### Diagnosis.

Very small, yellowish diving beetles (1.4–2.2 mm for the known species, 2.7 mm with the new one described below) with black markings on elytra. Shape of body globular to elongate, widest in middle, not flattened. 1) Occipital line present; 2) basal pronotal striae present; 3) basal elytral striae present; 4) sutural line on elytron absent; 5) basal epipleural transverse carina absent; 6) clypeus with fore margin narrowly and finely bordered, sometimes unmodified; 7) longitudinal elytral carina on disc absent; and 8) parameres two-segmented.

Includes 27 species (Nilsson 2016), widely distributed in the Aftrotropical, Oriental and Palearctic regions.

### Leiodytes kualalipis

Taxon classificationAnimaliaColeopteraDytiscidae

Balke, Wang, Bergsten & Hendrich sp. n.

http://zoobank.org/3F824AE1-26C9-4E15-AB50-5974F10ED08A

#### Holotype.

Male (ZSM). “Malaysia, Pahang, Kuala Lipis, old rubber plantation, iv.1997, Balke & Hendrich”. **Paratypes**, 2 males (CSR, ZSM), 1 female (CSR) “S Vietnam (Cat Tien), 120 km NNE Ho Chi Minh, Cat Tien NP, 3.–15.07.1995, A. Napolov leg.”; 2 males (CWT) “Vietnam, Nam Cat Tien, 200 m, 17-25-VI-1995, leg. Malicky”; 3 males, 2 females (CWT, NHMW, TFRI). “Vietnam, Dong NAI, Nam Cat Tien NP, 120 m, 18-IX-1998, leg. L. J. Wang”.

#### Type locality.

The type locality was around 4.200104°N 102.061570°E, altitude 100m. The entire region is now heavily transformed into oil palm plantations. The paratype locality was in Nam Cat Tien National Park, as a rough reference we obtained a position at the forest border 11.422096°N 107.427578°E.

#### Description.

Habitus elongate oval. Measurements: TL = 2.7 mm; TL-H = 2.4 mm; MW = 1.3 mm; elytra 4.2× longer than pronotum.

**Figure 3.**
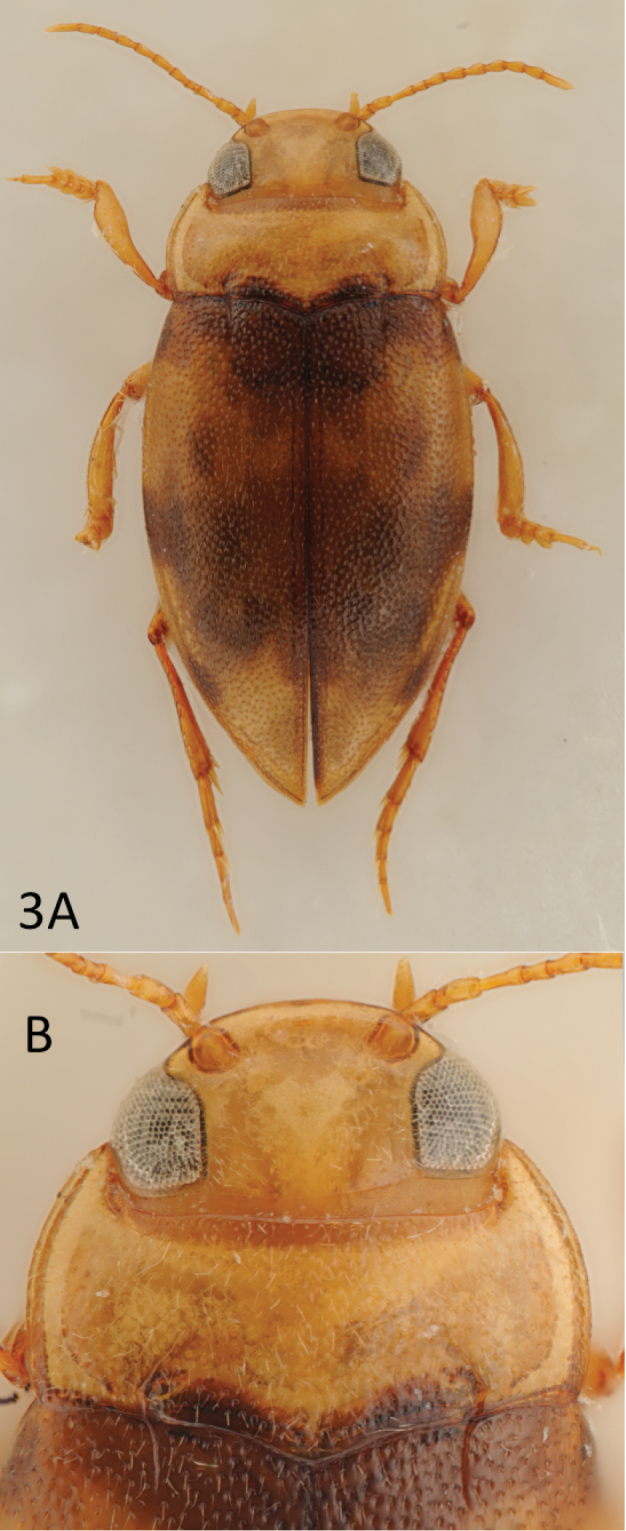
*Leiodytes kualalipis* sp. n., holotype, habitus (**A**), head, pronotum and base of elytra (**B**).

**Colouration.** Beetle dark yellow with few darker basal pronotal markings and darker pattern on elytra (Fig. 3).

**Surface sculpture.** Head with distinct microreticulation posterior of occipital line; faint to extremely faint microreticulation along eyes and clypeus, frons without microreticulation and thus shiny / polished but with few setiferous punctures. Pronotum and elytron without microreticulation but with dense, coarse setiferous punctation. Ventral surfaces mostly polished, metaventrite, metacoxa and ventrites 1–3 with large punctures bearing short setae, distance between punctures approximately the diameter of a single puncture (similar to *Leiodytes vietnamensis* Wang, Satô & Yang, 1998, p. 165, Fig. 6A in their work).

**Figure 4.**
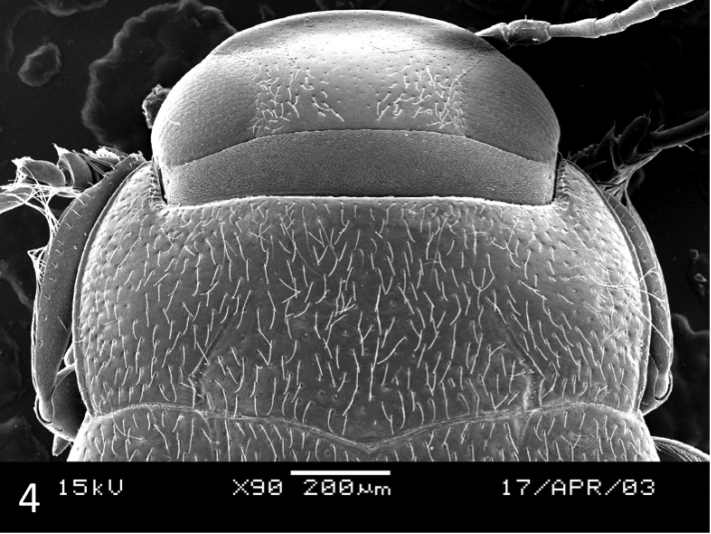
*Leiodytes kualalipis* sp. n.: male paratype, SEM of head, pronotum and base of elytra.

**Structures.** Head with faint occipital line and depressed before clypeus leading to the impression that the clypeus is broadly beaded (Figs 3B, 4). Pronotum with faint lateral bead and distinct basal striae, the latter are strongly directed inwards (Figs 3B, 4). Elytron with basal striae but without sutural lines. Basal epipleural transverse carina absent. Flight wings apparently fully developed (not dissected but seen from opening caused by previous removal of the last three ventrites). Pro- and mesotarsus not conspicuously dilated laterally.

**Male.** Median lobe of aedeagus in lateral view thin and pointed apically, apical portion spatulate in ventral view (Figs 7A, B); lateral lobes (parameres) of two parts and distally broad, with broad “nose” or broadly twisted (Fig. 7C).

**Figure 5.**
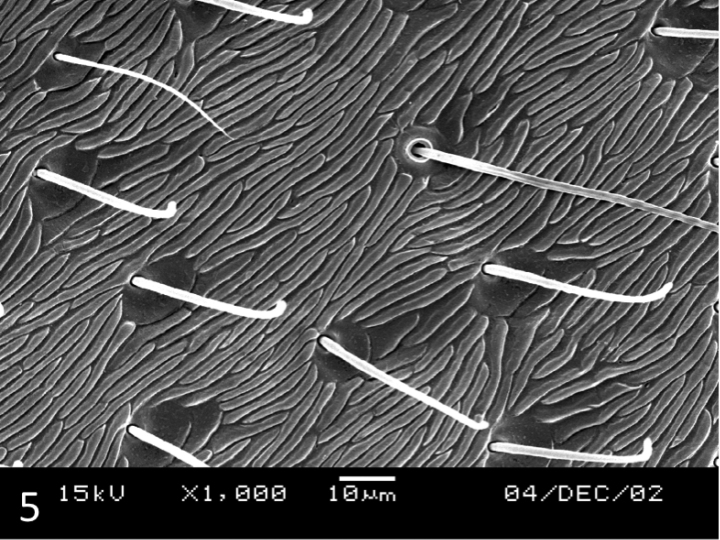
*Leiodytes kualalipis* sp. n.: female paratype, SEM of microreticulation on metasternum.

**Figure 6.**
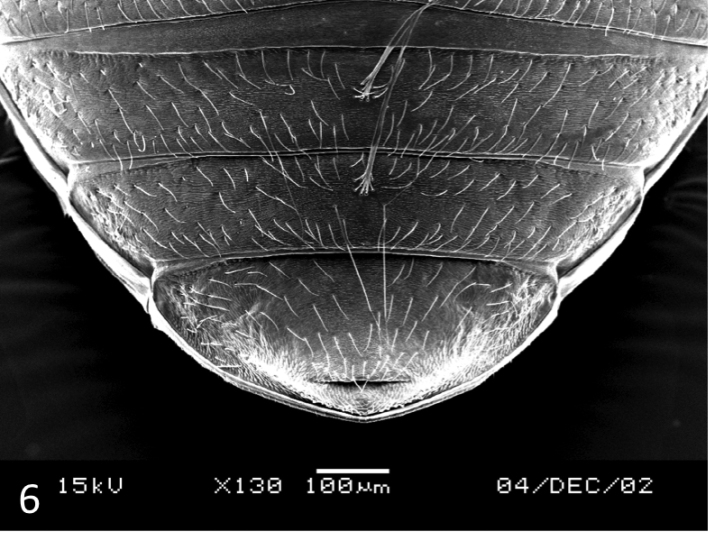
*Leiodytes kualalipis* sp. n.: female paratype, SEM of last ventrite with deep suture apically.

**Female.** Similar to male, but surface dull due to presence of strong microreticulation dorsally and ventrally (Figs 5, 6).

**Figure 7.**
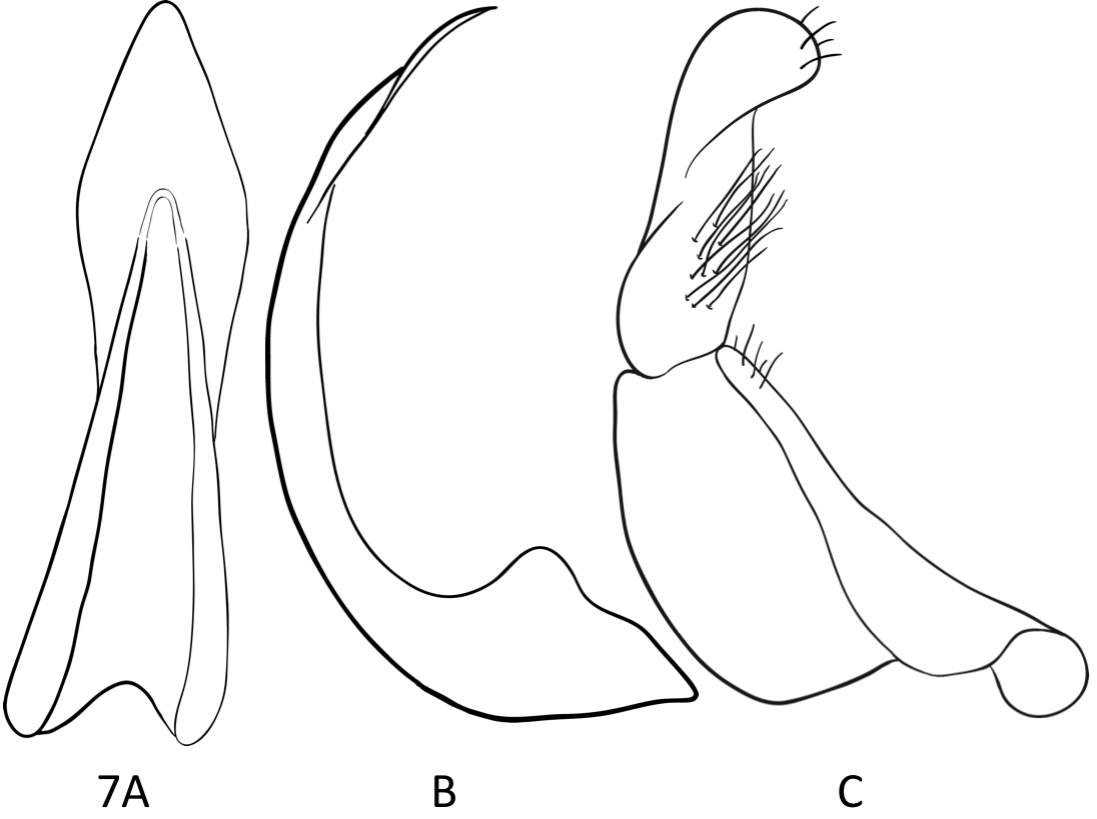
*Leiodytes kualalipis* sp. n., median lobe of aedeagus, ventral view (**A**), lateral view (**B**), paramere, lateral view (**C**).

#### Diagnosis.

This is the largest species of *Leiodytes* and well characterised by its size as the other species in the region are distinctly smaller (below 2.2 mm or even below 2 mm long, see e.g. Régimbart 1899; Wang et al. 1998). Besides the larger size, this new species has a unique feature: the last ventrite has a deep suture apically (Fig. 6). This might be an autapomorphy for the new species.

This is, to our knowledge, the second species reported from the Malayan Peninsula, *Leiodytes nicobaricus* (Redtenbacher, 1867) being the other one (Balke et al. 2004; Hendrich et al. 2004).

#### Etymology.

Named after the type locality, Kuala Lipis Town. The species name is a noun in apposition.

#### Distribution.

A species with a wide geographic range, known from the type locality in West Malaysia as well as from Southern Vietnam. The distance between these two localities is roughly 1,000 km measured as a straight line across the Gulf of Thailand.

#### Habitat.

**Figure 8.**
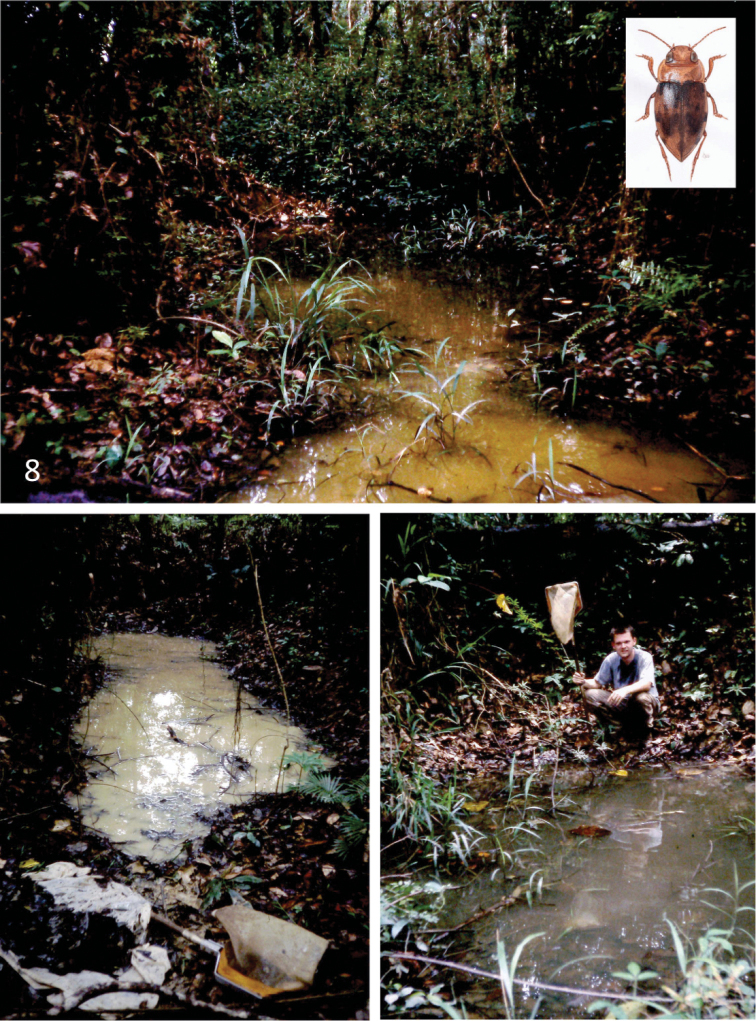
Habitat of *Leiodytes kualalipis* sp. n. near Kuala Lipis, West Malaysia. Drawing of beetle by D. Paramonov (Riga).

The holotype was collected in shallow water, among dense layers of rotten leaves, in a shaded forest pool situated in an old rubber plantation overgrown by secondary forest (Fig. 8). The species was associated with the Dytiscidae: *Copelatus* sp., *Hydaticus pacificus pacificus* Aubé, 1838 and *Hyphydrus birmanicus* Régimbart, 1888.

In Vietnam, the species was collected in shallow water made by jeep on a path. Syntopically occurring dytiscid species included *Leiodytes nicobaricus* (Redtenbacher, 1867), *Hydroglyphus orientalis* (Clark, 1863) and *Sandracottus maculatus* (Wehncke, 1876).

### Note

Nilsson (2016) in the updated World Catalogue of Dytiscidae lists two Bidessini species of unclear generic placement. One of them is *Hydroporus aberrans* Clark, 1863: 426, described from Indonesia (“Java”). The holotype cannot be located in the Natural History Museum London, which houses Clark’s collections. Clark (1863) wrote that he only received one imperfect specimen from Mr. Bowring from Java. It was about 1.6 mm long, with parallel-sided “abdomen” but laterally rounded thorax (narrow anteriorly and posteriorly) and each elytron had eight rows of coarse punctures (Clark referred to them as “striae”). The dorsal colouration was testaceous, with the area between the 1^st^–6^th^ rows being irregularly rufo-testaceous. This might refer to a species of *Leiodytes*, although we have not seen any species of that genus with rows of coarse serial punctures on the elytron.

We can however rule out that any of the new species proposed herein is conspecific with *Hydroporus aberrans*.

The other species of unclear generic placement was *Hydroporus fairmairei* Branden, 1885, which we transfer to *Limbodessus* below.

### New combinations and synonymies

#### Limbodessus curviplicatus

Taxon classificationAnimaliaColeopteraDytiscidae

(Zimmermann, 1927)

Fig. 9A, B

Bidessus curviplicatus Zimmermann, 1927:16 (Samoa).
Limbodessus curviplicatus (Zimmermann, 1927): Balke and Ribera 2004: 125.
= Hydroporus dorsoplagiatus Fairmaire, 1881: 249 (Fiji); preoccupied by Fairmaire (1880: 247), **syn. n.**
= Hydroporus fairmairei Branden, 1885: 53 (a replacement name for Hydroporus dorsoplagiatus Fairmaire, 1881); this replacement name is in turn preoccupied by Hydroporus fairmairei Leprieur (1876: 142, currently in Deronectes), objective synonym of Hydroporus dorsoplagiatus Fairmaire, 1881.
= Bidessus fijiensis J. Balfour-Browne, 1944: 99 (Fiji), **syn. n.**
Liodessus fijiensis (J. Balfour-Browne, 1944): Biström 1988: 19.
Limbodessus fijiensis (J. Balfour-Browne, 1944), **comb. n.**

##### Type material.

*Hydroporus dorsoplagiatus*: not located. A loan request was sent to MNHN with request number 66649 on 11.08.2016, and Antoine Mantilleri as well as later Dr. Arnaud Faille searched the collection and did not find potential type material. This might still be stored in the Oberthür collection, however, and might be found at some stage.

*Bidessus curviplicatus*: Holotype, male (BMNH) and paratype, male (ZSM), Samoan Is. Mulifanua Upolu, F. Burton and G.H. Hopkins, Type.

*Bidessus fijiensis*: Holotype, female (BMNH) Namaka, C Fiji 1545, 8.12.43, R.A. Lever, Pres. By Imp. Inst. Ent. B.M. 1945.9, Bidessus fijiensis Type! J. Balfour-Browne det.

##### Additional material.

8 exx (ZSM) Fiji: Viti Levu, Rakiraki, Navara, 50m, 10.xi.2003, 17.416024°S 178.147712°E, Wewalka & Balke (FI 11).

##### Diagnosis.

**Figure 9.**
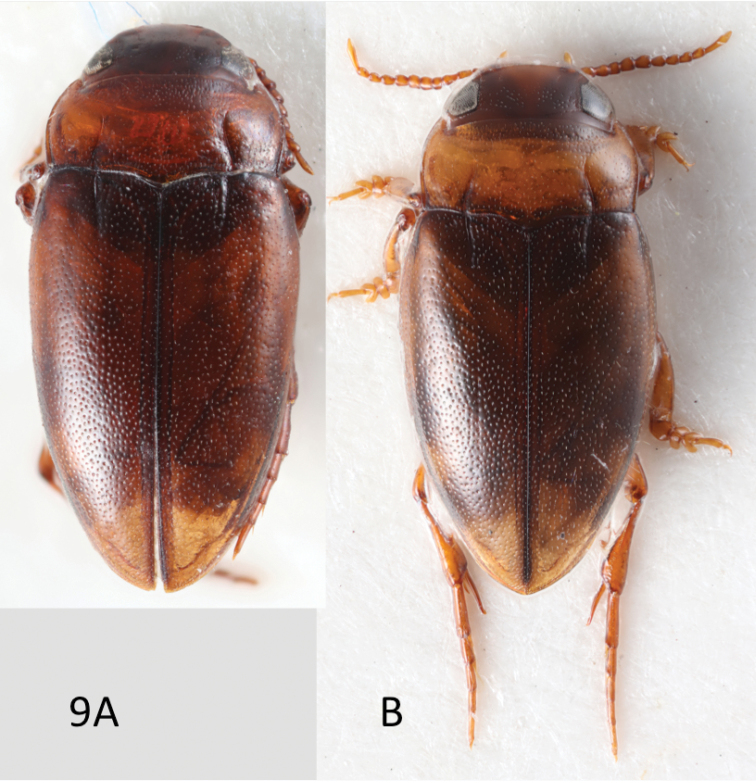
*Limbodessus curviplicatus*: male paratype of *Bidessus curviplicatus* Zimmermann, 1927 from Samoa (**A**) and male specimen from Fiji (**B**).

A stout, yellow to yellow orange *Limbodessus* with slightly enlarged male antenna and distinct angle between base of elytron and base of pronotum (Fig. 9). Female with strongly expanded antennomeres 3–6. Fijian specimens are slightly smaller than the Samoan types: 3.5 mm (Samoa) *versus* 2.8–3.2 mm (Fiji).

##### Notes on classification.

We have not seen type material of *Hydroporus dorsoplagiatus* but have collected specimens in Fiji that match the description by Fairmaire (1881). At the same time, we found that our Fiji specimens agree with the types of *Limbodessus curviplicatus* (Zimmermann, 1927) from Samoa. Moreover, *Bidessus fijiensis* J. Balfour-Browne, 1944, later moved to *Liodessus* by Biström (1988), and which we here move to *Limbodessus*, agrees well with our newly collected Fijian specimens and thus establishes another junior synonym to *Hydroporus fairmairei*, the replacement name of *Hydroporus dorsoplagiatus*. However, since the replacement name *Hydroporus fairmairei* suggested by Branden (1885) is also a junior homonym (see above), the oldest valid synonym, *Limbodessus curviplicatus* (Zimmermann, 1927), becomes the valid name.

##### Distribution.

Samoa, Fiji.

## Supplementary Material

XML Treatment for Rompindessus

XML Treatment for Rompindessus jenisi

XML Treatment for Leiodytes

XML Treatment for Leiodytes kualalipis

XML Treatment for Limbodessus curviplicatus
